# Modulation of yeast telomerase activity by Cdc13 and Est1 *in vitro*

**DOI:** 10.1038/srep34104

**Published:** 2016-09-23

**Authors:** Yu-Fan Chen, Chia-Ying Lu, Yi-Chien Lin, Tai-Yuan Yu, Chun-Ping Chang, Jing-Ru Li, Hung-Wen Li, Jing-Jer Lin

**Affiliations:** 1 Institute of Biopharmaceutical Sciences, National Yang-Ming University, Taipei 112, Taiwan; 2 Institute of Biochemistry and Molecular Biology, National Taiwan University College of Medicine, Taipei 100, Taiwan; 3 Department of Chemistry, National Taiwan University, Taipei 100, Taiwan

## Abstract

Telomerase is the enzyme involved in extending telomeric DNA. Control of telomerase activity by modulating its access to chromosome ends is one of the most important fundamental mechanisms. This study established an *in vitro* yeast telomerase reconstitution system that resembles telomere replication *in vivo*. In this system, a tailed-duplex DNA formed by telomeric DNA was employed to mimic the structure of telomeres. The core catalytic components of telomerase Est2/Tlc1 RNA were used as the telomeric DNA extension machinery. Using the reconstituted systems, this study found that binding of Cdc13 to telomeric DNA inhibited the access of telomerase to its substrate. The result was further confirmed by a single-molecule approach using the tethered-particle motion (TPM)-based telomerase assay. The findings also showed that the inhibitory effect can be relieved by telomerase-associated protein Est1, consistent with the role of Cdc13 and Est1 in regulating telomere extension *in vivo*. Significantly, this study found that the DNA binding property of Cdc13 was altered by Est1, providing the first mechanistic evidence of Est1 regulating the access of telomerase to its substrate. Thus, the roles of Cdc13 and Est1 in modulating telomerase activity were clearly defined using the *in vitro* reconstituted system.

Telomeres are the protein-DNA complexes at the termini of eukaryotic chromosomes. They are essential for the preservation and complete replication of chromosomes^1^. Telomerase is a ribonucleoprotein involved in telomere replication. It contains a catalytic protein component TERT (telomerase reverse transcriptase) and an associated RNA moiety TER, which serves as the template to extend telomeric DNA sequences. In *S. cerevisiae*, these two components are encoded by *EST2*^2^,^3^ and *TLC1*^4^, respectively. It was shown that Est2 and Tlc1 RNA are sufficient to reconstitute the core telomerase catalytic activity *in vitro*^5^. However, *in vivo* telomerase functions require three other proteins including Est1, Est3, and Cdc13. Mutations in *EST2, TLC1, EST1, EST3* and *CDC13* cause progressive shortening of telomeres that eventually lead to cell death, suggesting an essential role of these genes in telomere maintenance^6^,^7^. Est1 is associated with telomerase through binding to a bulged stem element of Tlc1 RNA^8^. Cdc13 is a single-stranded telomeric TG_1–3_ DNA-binding protein that interacts with telomeres^9^,^10^. Chromatin immunoprecipitation experiments also showed that both Est1 and Cdc13 are enriched at telomeres during the S phase, providing a timely support for its role in telomere length regulation^11^. The role of Est3 is less clear. It is enriched at telomeres in late S/G2 phase and binds to telomere in an Est1-dependent manner^12^. Since neither Est1 nor Est3 reveals a significant effect on telomerase activity *in vitro*, they are taken as regulatory factors for telomerase activity^5^.

Telomere-associated proteins have been implicated as a fundamental mechanism in regulating telomerase activity^13^. Indeed, it was shown that both Est1 and Cdc13 are involved in mediating telomerase extension by both genetic and protein fusion experiments^11^,^14^,^15^,^16^,^17^,^18^. The mechanism of how Cdc13-Est1 mediated telomere extension by telomerase has been previously analyzed. Two models were put forward to describe the role of Cdc13 and Est1 in this process. The first model proposes that Est1 forms complex with the Est2/Tlc1 telomerase and then recruits to telomere through interacting with Cdc13 in late S phase of the cell cycle. This model was supported by both two-hybrid and biochemical analyses on the interaction between Cdc13 and Est1^15^, a series of fusion and suppression analyses^14^,^18^,^19^, and detailed chromatin-immunoprecipitation studies^20^. Live cell imaging of single telomerase particles also provided evidence that telomerase associates with telomeres only in late S phase^21^. Moreover, addition of newly synthesized telomeres onto double-strand breaks appears to require Cdc13-Est1 interaction to bring telomerase to its site of action^18^, further supporting the recruiting model. The second model was first put forward after a series of chromatin-immunoprecipitation studies^11^. It proposed that telomerase complex is in a telomere-associated and inactive state during most stages of the cell cycle. Accumulation of Est1 at late S phase of the cell cycle then activates the activity of telomere-bound telomerase. However, the activation model is not supported by the observation that the Est1-Est2/Tlc1 RNP complex was formed before loading onto telomeres^22^,^23^. Currently, there is no biochemical evidence to further support the activation model.

Although genetic analyses have established a role of Cdc13 and Est1 in modulating telomerase activity, the detailed mechanism is not clearly defined. Using telomerases reconstituted from an *in vitro* translation system and an overexpressing system, Zappulla *et al*. reported that Cdc13 inhibited telomerase activity^24^. On the contrary, DeZwaan *et al*. found that Cdc13 activated telomerase activity and the telomeric DNA binding activity of Cdc13 was not required for the activation^25^. It was further shown by DeZwaan *et al*. that Est1 activated telomerase activity and both Cdc13 and Est1 cooperatively achieved a combinatorial activation effect on telomerase^26^. Although the apparent discrepancy may be explained by the ways telomerase were prepared, how Cdc13 and/or Est1 modulate telomerase is not fully addressed. The detailed mechanistic of telomerase activity modulated by Cdc13 and Est1 remains unclear.

The present study established an *in vitro* reconstituted system to analyze the mechanism of how Cdc13 and Est1 regulate telomerase activity. Using both biochemical and single-molecule approaches, this study found that binding of Cdc13 to telomeric DNA inhibits telomerase activity. The DNA binding activity of Cdc13 is required for inhibition; hence, the apparent inhibitory effect is most likely caused by blocking the access of telomerase to its substrate. Although Est1 alone does not have a direct effect on telomerase activity, it relieves the telomerase-inhibitory effect of Cdc13. The interaction between Cdc13 and Est1 is important for modulating telomerase activity. Moreover, this study found that Est1 alters the DNA-binding properties of Cdc13. Taken together, the results provide a molecular mechanism on how Est1 relieves the telomerase inhibitory effect of Cdc13. The present findings are consistent with the roles of Cdc13 and Est1 *in vivo* and clearly define their involvement in regulating telomerase activity.

## Results

### Establishing an *in vitro* telomerase analysis system using Est2/Tlc1 RNA complexes and a tailed-duplex DNA

**Figure 1:**
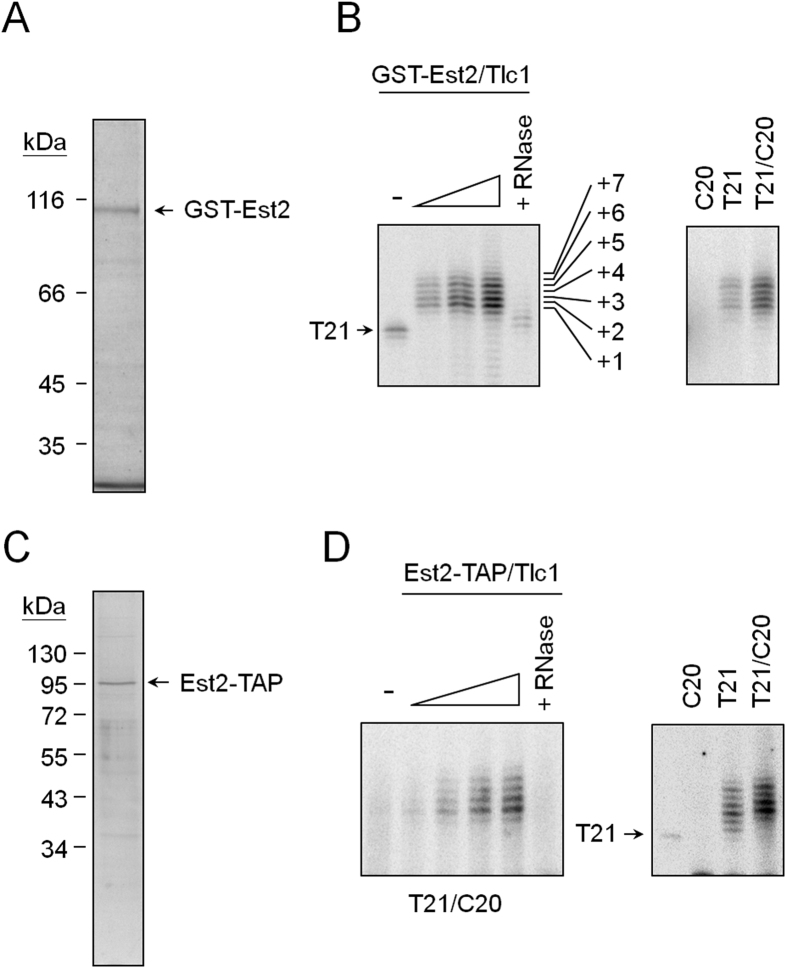
*In vitro* reconstitution of yeast telomerase. (**A**) Purification of GST-Est2/Tlc1 RNP. Yeast BJ2168 cells carrying plasmids pEG(KT)-EST2 and pRS424-GAL-TLC1 were induced by growing the cells in galactose medium and then purified using glutathione sepharose. The isolated complexes were separated by an 8% SDS-polyacrylamide gel and stained by silver. Arrow indicates the position of GST-Est2. (**B**) The telomerase activity was analyzed by primer extension assay. Left: 30 nM of T21 DNA was incubated with 0.75, 1.5 or 3 ng of the isolated GST-Est2/Tlc1 RNP in the presence of [α-^32^P]dGTP and dTTP at 30 °C for 1 hr. The reaction products were then separated by a 12% sequencing gel. In lane 5, RNase A pretreatment of GST-Est2/Tlc1 RNP was used in the reaction. Right: 30 nM each of C20, T21, and T21/C20 DNA were incubated with 1 ng of GST-Est2/Tlc1 RNP and telomerase activity analysis was then conducted. (**C**) Isolation and telomeric DNA extension activity of Est2-TAP/Tlc1 RNP. The C-terminal TAP-tagged Est2/Tlc1 RNP was isolated and analyzed by a 10% SDS-polyacrylamide gel and stained by silver. (**D**) The telomerase activity was analyzed by primer extension assay. Left: 30 nM of T21 DNA was incubated with 0.37, 0.75, 1.5 or 3 ng of the isolated Est2-TAP/Tlc1 RNP and analyzed as in Fig. 1B. Right: 30 nM each of C20, T21, and T21/C20 DNA were incubated with 1 ng of Est2-TAP/Tlc1 RNP and telomerase activity analysis was then conducted.

This study chose a yeast strain over-expressing both GST-tagged Est2 and Tlc1 RNA as the initial source of telomerase activity^27^. The core telomerase activity formed by GST-Est2 and Tlc1 RNA, a ribonucleoprotein (RNP) complex, could then be isolated using glutathione-sepharose resins. As shown in Fig. 1A, both GST-Est2 and Tlc1 RNA were readily detected in the isolated complexes (also in Fig. S1). Cdc13 and Est1 were not detected in the isolated complexes as judged by the immunoblotting experiments using polyclonal antibodies raised against these two proteins (data not shown). The isolated GST-Est2/Tlc1 RNP could utilize yeast single-stranded telomeric DNA T21 as substrate to extend up to seven-nucleotide telomeric sequences (Fig. 1B, left panel). Extension of telomeric DNA by GST-Est2/Tlc1 RNP was sensitive to RNase A digestion, suggesting that the extended products were indeed derived from telomerase activity. Thus, the isolated GST-Est2/Tlc1 RNP had activity consistent with the previous reports of telomerase isolated from the reconstituted enzyme^27^ or directly from yeast^28^.

A tailed-duplex DNA substrate was then designed by annealing T21 and C20 oligonucleotides for the analysis of this study (Materials and Methods). The resulting DNA mimics the structure of telomeres. It has a six-base-pair telomeric DNA duplex and a 15-nucleotide single-stranded G-tail on one end. The C20 complementary strand was also designed to have an amino group at the 3′ end to prevent non-specific extensions. As shown in Fig. 1B (right panel), the C20 DNA alone cannot be extended by GST-Est2/Tlc1 RNP. As expected, the tailed-duplex DNA (T21/C20) could be effectively recognized by GST-Est2/Tlc1 RNP as a substrate. It also appeared that the tailed-duplex DNA was a better substrate for Est2/Tlc1 RNP.

As an additional source of telomerase, this study also used yeast cells over-expressing C-terminal TAP (tandem affinity purification)-tagged Est2 (Est2-TAP) and Tlc1 RNA to isolate telomerase complex. Est2-TAP/Tlc1 RNP was then purified by stepwise binding-and-elusions using IgG and calmodulin beads. As shown in Fig. 1C, Est2-TAP was the major protein in the isolated fraction. The isolated Est2-TAP/Tlc1 RNP readily extended telomeric DNA substrates (Fig. 1D, left panel) and appeared to prefer the tailed-duplex DNA as substrate (Fig. 1D, right panel). These findings revealed an *in vitro* telomerase assay system established successfully using isolated GST-Est2/Tlc1 RNP and Est2-TAP/Tlc1 RNP complexes and a tailed-duplex DNA to mimic telomerase extension *in vivo*.

### Cdc13 inhibits telomerase activity through limiting telomerase accesses to telomeric DNA

**Figure 2:**
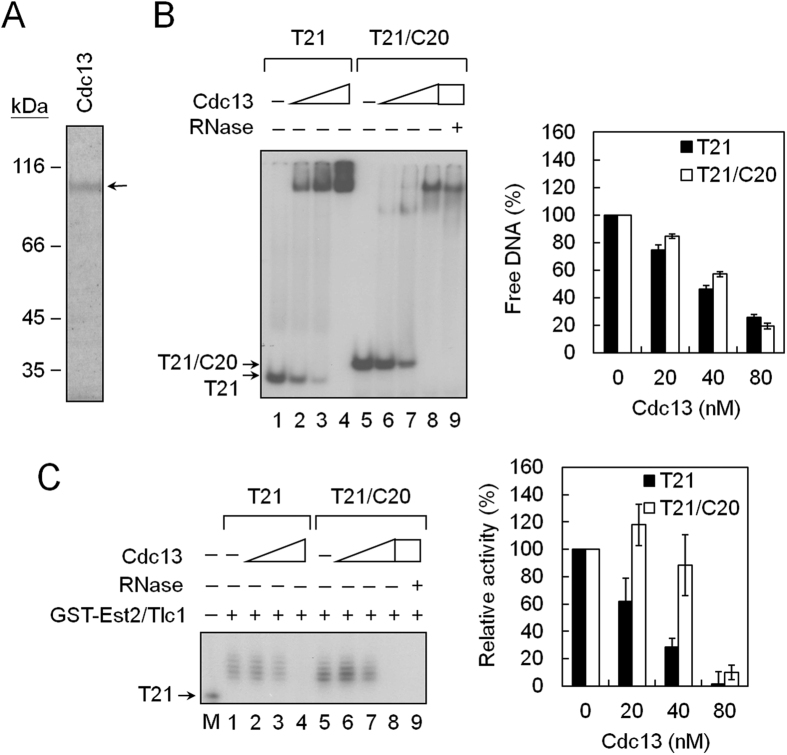
Cdc13 inhibits telomerase activity. (**A**) Purification of Cdc13. Cdc13 with 6xHis tag were purified from sf21 using a Ni-NTA agarose column. One μg of purified Cdc13 was separated by a 10% SDS polyacrylamide gel. A Coomassie blue-stained gel is given. (**B**) The single-stranded T21 DNA and tailed-duplex DNA binding activity of Cdc13. Thirty nM of ^32^P-labeled T21 or T21/C20 DNA (labeled on T21) was mixed with 20, 40, or 80 nM of purified Cdc13 at room temperature for 5 min and analyzed by EMSA. An image of the gel taken by PhosphorImager (left) and the quantification of the binding activity (right) is presented. (**C**) Effect of Cdc13 on telomerase activity of GST-Est2/Tlc1 RNA complexes. Thirty nM of T21 or T21/C20 DNA was incubated with 20, 40, or 80 nM of purified Cdc13 at room temperature for 5 min. GST-Est2/Tlc1 RNP was then added to the reaction mixtures and the telomerase activity was analyzed by primer extension assay. An image of the gel taken by PhosphorImager (left) and the quantification of the telomease activity (right) is presented. M shows the position of primer T21 DNA as a size maker. In lane 9, GST-Est2/Tlc1 RNP was pretreated with RNase A at 30 °C for 10 min.

**Figure 3:**
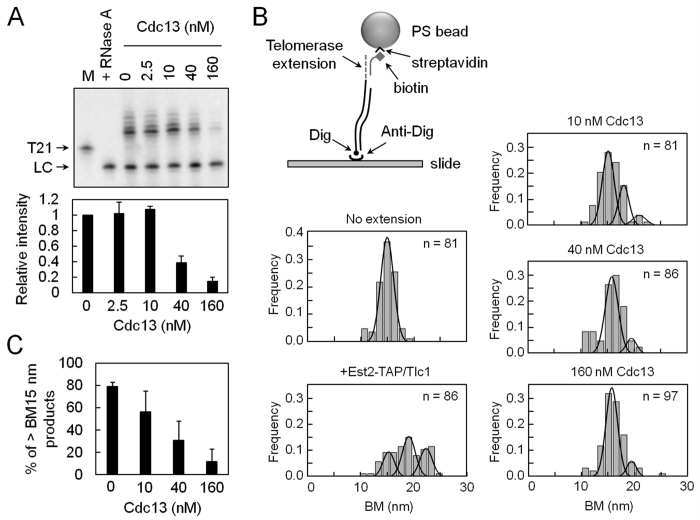
Single-molecule TPM experiments for telomerase inhibition by Cdc13. (**A**) Effect of Cdc13 on telomerase activity of Est2-TAP/Tlc1 RNP. Thirty nM of T21/C20 DNA was incubated with 2.5, 10, 40, or 160 nM of purified Cdc13 at room temperature for 5 min. Est2-TAP/Tlc1 RNP was then added to the reaction mixtures and the telomerase activity was analyzed by primer extension assay. An image of the gel taken by PhosphorImager (top) and the quantification of telomease activity (bottom) are presented. M shows the position of primer T21 DNA as a size maker. In RNase lane, the Est2-TAP/Tlc1 RNP was pretreated with RNase A at 30 °C for 10 min. (**B**) Single-molecule TPM method for monitoring telomerase activity inhibited by Cdc13. Digoxigenin (Dig)-labeled DNA substrates were incubated with 4 nM Est2-TAP/Tlc1 RNP at 30 °C. The reaction products were stopped by Proteinase K and RNase H and then immobilized in glass surface by anti-digoxigenin (anti-Dig) antibody. The extended DNA were then annealed with biotin-labeled oligonucleotide probes and then with streptavidin-coated polystyrene beads for TPM analysis. BM histograms of DNA substrates without telomerase incubation, extended by Est2-TAP/Tlc1 RNP, and incubated with 10, 40, or 160 nM Cdc13 are presented. (**C**) The percentage of BM peaks with values exceeding 15 nm decreases with increasing Cdc13 concentration.

To evaluate the effects of telomere-binding protein Cdc13 on telomerase activity, Cdc13 was isolated using a baculovirus system (Fig. 2A)^29^. In parallel experiments monitoring both DNA-binding and telomerase-inhibition activities of Cdc13 using EMSA and primer extension assays, respectively, Cdc13 was found to bind to both single-stranded telomeric DNA and the tailed-duplex DNA (Fig. 2B). The levels of Cdc13-binding and telomerase-inhibition were well correlated when single-stranded T21 DNA was used as a substrate (Fig. 2B,C). Interestingly, slightly higher telomerase activity was observed at low concentration of Cdc13 on the tailed-duplex T21/C20 DNA, although telomerase activity was inhibited when most of the DNA was bound by Cdc13 (Fig. 2C). The inhibitory effect of Cdc13 was further analyzed using Est2-TAP/Tlc1 RNP. As shown in Fig. 3A, Cdc13 effectively inhibited DNA extension activity of Est2-TAP/Tlc1 RNP, with minimal telomerase activation effect observed at low concentration of Cdc13. Thus, the current biochemical results obtained using two different isolates of telomerases showed that Cdc13 inhibited telomerase activity. The biological implication of Cdc13 activating telomerase at low concentration remains unclear.

Conventional primer extension assay monitors the products of telomerase-mediated telomere extension using the gel electrophoresis technique^28^. However, owing to the sensitivity of gel assays and the amount of DNA required, the assay detects mostly single-round or double-rounds DNA extension activity by yeast telomerases. A single-molecule telomere extension assay involving tethered particle motion (TPM) has previously been developed for monitoring multiple rounds of telomerase-mediated telomere extension^30^. This study adopted the TPM-based assay as an independent approach to monitoring the effect of Cdc13 on telomerase.

In the TPM assay, a 5′-digoxigenin labeled tailed-duplex DNA substrate was first incubated with Est2-TAP/Tlc1 RNP to allow extension of telomeric DNA sequences (Fig. 3B). The extended products were immobilized onto the anti-digoxigenin-coated slide surface, and were then annealed to polystyrene (PS) beads coated with oligonucleotide probes. These individually tethered DNA-beads underwent Brownian motion (BM), which could be visualized under an optical microscope. BM distributions were then analyzed. A single BM peak at ~15 nm represented DNA products of no or ‘single-round’ extension (Fig. 3B, no extension). Larger BM peaks were observed when the DNA substrates were extended by telomerase (Fig. 3B, +Est2-TAP/Tlc1). Increased population of higher BM peaks as well as more peaks in higher BM indicated that telomerase was capable of extending multiple rounds of reactions on the same DNA substrates. The percentage of high BM peaks disappeared gradually with increase in Cdc13 added (Fig. 3B,C). Thus, the single-molecule telomerase assay demonstrated directly inhibition of telomerase by Cdc13. In view of the results from both biochemical and single-molecule analyses, we concluded that Cdc13 inhibits telomerase *in vitro*. It is also interesting to note that since the telomerase-inhibitory effects correlated well with the binding of Cdc13 onto DNA substrates, these results also suggested that Cdc13 inhibited telomerase activity through its binding to DNA substrate.

**Figure 4:**
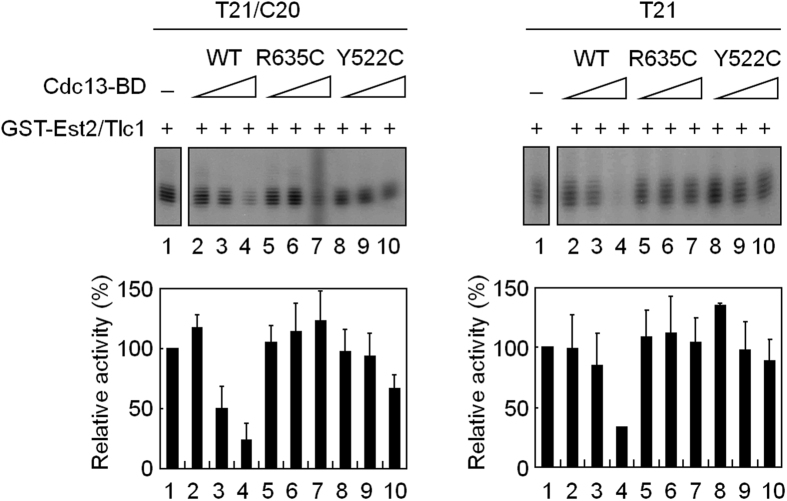
Telomere-binding activity of Cdc13 is required for inhibition of telomerase activity. T21/C20 (left) or T21 (right) DNA was mixed with 20, 40, or 80 nM of purified Cdc13-BD proteins at 30 °C for 5 min. The mixtures were then mixed with GST-Est2/Tlc1 RNP and then analyzed by primer extension assay. Images of the gels taken by PhosphorImager (top) and the quantification of telomerase activities (bottom) are presented.

To determine how Cdc13 inhibits telomerase activity, this study examined whether the DNA binding activity of Cdc13 is required for telomerase inhibition. Previous studies had identified two mutants, *cdc13*^*R635C*^ and *cdc13*^*Y522C*^, that had lost the telomere-binding activity^31^. The telomere-binding domain, Cdc13-BD, of wild-type and two mutants were isolated (Fig. S2A). In parallel experiments conducted with both EMSA and primer extension assays, these two mutants failed to bind to telomeric DNA or inhibit telomerase activity (Fig. 4 and S2B). Thus, the results indicated that Cdc13 inhibits telomerase through blocking the access of telomerase to its substrate.

### Est1 restores telomerase activity on Cdc13-bound telomeres

**Figure 5:**
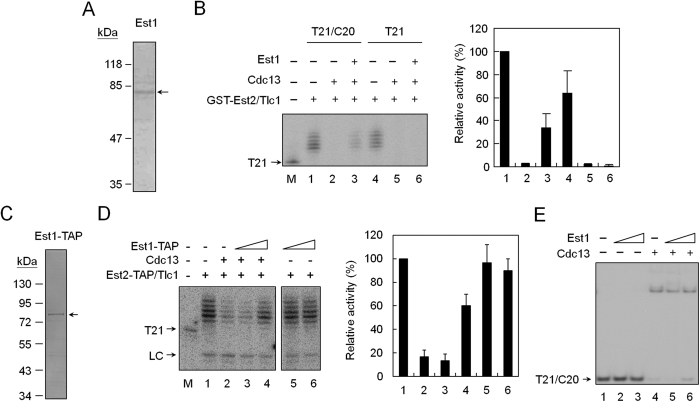
Activation of telomerase by Est1 on Cdc13-bound tailed-duplex DNA. (**A**) Purification of Est1 protein. Est1 was isolated from baculovirus-infected sf21 cells. One μg of purified proteins was separated by an 8% SDS polyacrylamide gel and stained with Coomassie blue. (**B**) Est1 restores telomerase on Cdc13-bound tailed duplex DNA. Thirty nM of T21/C20 DNA was pre-incubated with or without 80 nM Cdc13 at room temperature for 5 min. GST-Est2/Tlc1 RNP was then added to the reactions in the presence of 240 nM Est1. The telomerase activity was analyzed by primer extension assay. (**C**) Purification of TAP-tagged Est1 protein. Est1-TAP was isolated from yeast cells carrying plasmid pRS426-GAL-EST1-TAP. One μg of purified proteins was separated by a 10% SDS polyacrylamide gel and stained with Coomassie blue. (**D**) Activation of telomerase activity by Est1-TAP on Cdc13-bound tailed-duplex DNA. Thirty nM of T21/C20 DNA was pre-incubated with 80 nM of Cdc13 at 30 °C for 5 min. Est2-TAP/Tlc1 RNP was then added to the reactions in the absence or presence of 80 or 240 nM of Est1-TAP. The telomerase activity was analyzed by primer extension assay. Images of the gels taken by PhosphorImager are presented. (**E**) As above, the extent of DNA binding by Cdc13 and Est1 was analyzed by EMSA. The amounts of Est1 used were 80 and 240 nM, respectively.

Although Est1 is not required for the catalytic activity of telomerase reconstituted *in vitro*^5^, it clearly has a role in mediating telomere extension by telomerase *in vivo*^14^. The role of Est1 in regulating telomerase activity on Cdc13-bound telomeres was evaluated using the *in vitro* reconstituted system established in this study. Here Est1 was expressed and isolated from insect cells using the baculovirus expression system (Fig. 5A). The purified Est1 was then tested for its effect on telomerase activity. Est1 was found to be capable of restoring telomerase activity on Cdc13-bound tailed-duplex DNA (Fig. 5B). Up to 30–40% of telomerase activities were restored when Est1 was added to the Cdc13-bound tailed-duplex DNA. Interestingly, Est1 failed to restore telomerase activity when single-stranded telomeric DNA was used as substrate. Thus, the duplex portion within the tailed-duplex DNA might also have a role for Est1 to restore telomerase activity. As an additional proof, Est1 was prepared using TAP-tagged expression and isolation system (Fig. 5C). Similarly, Est1-TAP was found to effectively restore telomerase activity that was inhibited by Cdc13 (Fig. 5D) and Est1-TAP alone did not affect telomerase activity. The status of Est1-TAP binding to DNA substrate and its effect on telomerase activity in the presence or absence of Cdc13 were also evaluated. As shown in Fig. 5E, under the condition that Est1-TAP restored telomerase activity on Cdc13-bound DNA, Est1-TAP alone did not form stable protein-DNA complex with the substrate DNA. Interestingly, Est1-TAP slightly decreased the DNA-binding activity of Cdc13.

Although isolation of recombinant Est1 protein has been reported by several groups^25^,^32^,^33^,^34^, an issue concerning Est1 protein is its preparation and stability. We had tried to express and isolate recombinant Est1 from *E. coli* using various constructs. However, none of the constructs enabled successful isolation of Est1. We then applied both baculoviral and yeast expression systems to successfully express Est1 for this study. To prevent protein aggregations, we isolated Est1 using buffers containing 20% glycerol. We also frozen aliquots of the purified Est1 by dry ice/ethanol bath and stored them at −80 °C to minimize the risk of denaturing Est1 protein. Freshly thawed Est1 was used for experiments and was not frozen again to avoid disruption of protein structure by repeated freeze-thawing. Gel filtration column chromatography was also used to analyze if the purified Est1 forms large protein aggregations. Purified Est1-TAP (~82 kDa) eluted from the gel filtration column formed two distinct populations (Fig. S4). One population eluted with the mass ~160 kDa and the other population eluted at ~300–400 kDa. Judged by their sizes, it is likely that Est1 forms both dimer and tetramer in solution. The functional significance of the Est1 dimer-tetramer is not clear to us. Nevertheless, there is no Est1 eluted beyond 440 kDa, suggesting that there is no mis-folded protein aggregates in our Est1 preparations. It is also important to note that the isolated Est1s are capable of restoring the inhibitory effect of Cdc13, indicating that they are functionally competent.

### Interaction between Cdc13 and Est1 is required for proper activation of telomerase

**Figure 6:**
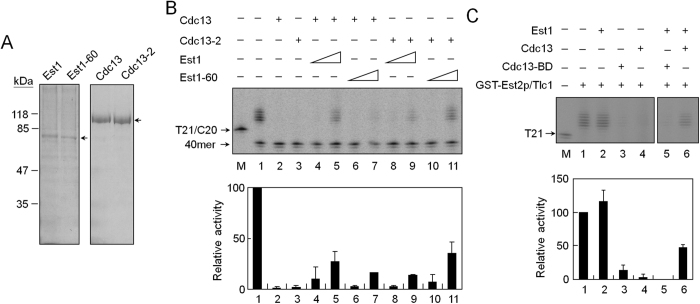
Interaction between Cdc13 and Est1 is required for activation of telomerase. (**A**) Purification of Est1-60 and Cdc13-2 proteins. Both proteins were expressed from baculovirus-infected sf21 cells and then purified by Ni-resin. One μg each of purified proteins was analyzed on an 8% SDS polyacrylamide gel. The Coomassie blue-stained gels are shown. (**B**) Effect of Est1-Cdc13 interaction on activation of telomerase activity. T21/C20 DNA was incubated with 80 nM Cdc13 or Cdc13-2 at 30 °C for 5 min. The mixture was then mixed with Est2/Tlc1 RNA complexes in the presence of 80 or 240 nM of Est1 or Est1-60. The telomerase activity was analyzed by primer extension assay. (**C**) Est1 cannot restore telomerase activity on Cdc13-BD-bound DNA. As above, 80 nM of Cdc13-BD was used in the analysis.

Previous studies identified two mutant alleles, *cdc13-2* and *est1-60*, that failed to support telomere extension *in vivo*^9^,^19^. As a result, both alleles showed progressive telomere shortening upon each cell division and eventually led to senescence. The *cdc13-2* allele changes the residue Glu252 to Lys whereas the *est1-60* allele has a reciprocal amino acid change at residue Lys444. Remarkably, *est1-60* was shown to be an allelic specific-mutant that suppressed the telomere defects of *cdc13-2.* Since telomerase extension function could be restored by reciprocal changes of amino residue, it was concluded that proper interaction between Cdc13 and Est1 is required for telomerase activity^19^. Thus, these two mutant alleles, *cdc13-2* and *est1-60*, were integrated into the analysis of this study. Both Est1-60 and Cdc13-2 were expressed and isolated using the baculovirus expression system (Fig. 6A). The purified Cdc13-2 bound to telomeric DNA with affinity similar to that of the wild-type protein (Fig. S3), indicating that the DNA-binding activity was not affected by this mutation^33^. These two mutant proteins were then analyzed for their effects on telomerase activity. Consistent with earlier observation that binding of Cdc13 to telomeric DNA inhibited telomerase, Cdc13-2 also inhibited telomerase activity (Fig. 6B, lane 3). However, the ability to restore telomerase activity in the presence of wild-type Est1 was significantly decreased (Fig. 6B, lanes 8–9). Similarly, the ability to restore telomerase on wild-type Cdc13-bound DNA by Est1-60 was also decreased (Fig. 6B, lanes 6–7). Remarkably, Est1-60 was capable of restoring telomerase activity on Cdc13-2-bound telomeres to a level similar to that by the wild-type proteins (Fig. 6B, lanes 10–11). Thus, the interaction between Cdc13 and Est1 was important for restoring telomerase on Cdc13-bound DNA.

To further support this conclusion, the DNA-binding domain of Cdc13, which does not interact with Est1, was used in the analysis. The DNA-binding domain of Cdc13 was capable of binding to telomeres and inhibiting telomerase activity (Figs 4 and 6C); however, it could not support Est1 to restore telomerase activity (Fig. 6C). Together, these results clearly demonstrated that interaction between Cdc13 and Est1 is crucial for telomerase on Cdc13-bound telomeres.

### Alteration of Cdc13 DNA binding property by Est1

**Figure 7:**
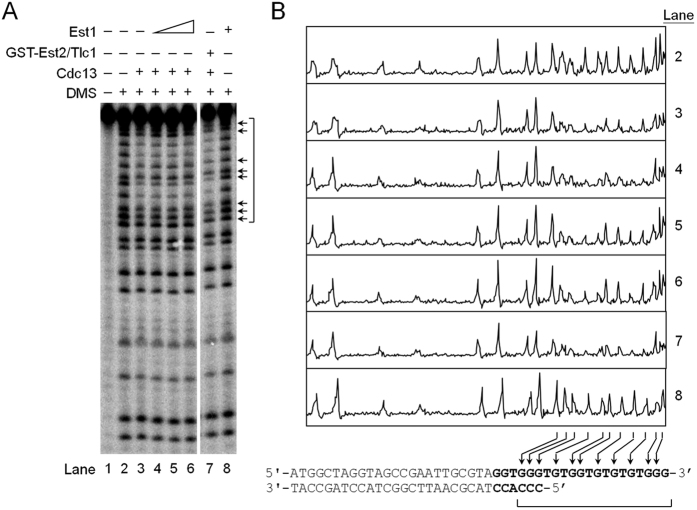
Alteration of Cdc13 DNA-binding property by Est1. (**A**) DMS footprint analysis of Cdc13 on tailed-duplex DNA. Tailed-duplex DNA was formed by annealing C20 and ^32^P-labeled T21. The DNA was incubated with 80 nM of Cdc13 at room temperature for 5 min. The mixture was then mixed with 100, 300, or 900 nM of Est1. The reaction products were then subjected to DMS footprint analysis using a 10% sequencing gel. An image of the gel taken by PhosphorImager is presented. Positions of guanines that are protected by Cdc13 are bracketed. In lanes 7 and 8, 3 ng of GST-Est2/Tlc1 RNP and 900 nM of Est1 were used in the analysis, respectively. (**B**) The radioactivity level of (**A**) was traced using a PhosphorImager, and the radioactivity profiles are plotted. Arrows indicate the positions of guanines that are affected by Est1. The sequence and the corresponding position of guanines on tailed-duplex DNA are also indicated. Region of telomeric DNA sequences is shown in bold font.

The mechanism of how Est1 restores telomerase activity on Cdc13-bound tailed-duplex DNA was further analyzed. Here, dimethyl sulfate (DMS) footprint assay was employed to better determine the Cdc13-telomeric DNA interactions. It enables the analysis of Cdc13-telomeric DNA interaction at single-nucleotide resolution. Cdc13 was first incubated with the tailed-duplex DNA substrate and Est1 was then added to the reactions. The results of Cdc13 footprints following DMS treatments were shown in Fig. 7A. The radioactivities of the footprints were also traced to reveal the differences between various treatments (Fig. 7B). It was apparent that Cdc13 protected a series of G-residues within the single-stranded region (Fig. 7A,B, lane 3). In addition, three G-residues located at the junction between the single-stranded and the duplex DNA were also protected. The results indicated that Cdc13 bound to both single-stranded residues and three G-residues within the double-stranded regions of the tailed-duplex DNA.

Results for DMS footprint experiments also showed that Est1 alone did not alter the pattern of cleavage induced by DMS/piperidine treatment (Fig. 7A, compare lanes 2 and 8). Significantly, addition of Est1 onto Cdc13-bound DNA caused a marked change in Cdc13-footprint. The Cdc13-protected G-residues were exposed to DMS upon the addition of Est1 in a dose-dependent manner (Fig. 7B, indicated by arrows). The results indicated that addition of Est1 altered the overall DNA-binding property of Cdc13 and exposed the 3′ end of the DNA. It is also important to note that addition of telomerase into Cdc13-bound DNA did not cause a major change in Cdc13 footprints (Fig. 7, lane 7), indicating that telomerase alone does not alter the telomeric DNA-binding properties of Cdc13. Together, the current findings indicated that the telomeric DNA-binding property of Cdc13 was altered by Est1.

## Discussion

Telomerase activity is regulated by Telomere-associated proteins. The current *in vitro* analyses showed that Cdc13 limits the access of telomerase to its substrate in a telomeric DNA-binding-dependent manner. The results provide the first level of regulating telomerase activity through controlling the accessibility of telomere end. Est1 alone does not affect telomerase activity; it regulates telomerase through the interaction with Cdc13^15^. This study showed that Est1-Cdc13 interaction is required for telomerase to extend telomere end, supporting the general concept of Cdc13-Est1 interaction in recruiting telomerase onto telomeres^14^,^15^,^18^,^19^. Moreover, the DMS footprint analyses showed that Est1-Cdc13 interaction changes the DNA binding property of Cdc13 to expose the telomeric end for telomerase extension. The results provide a mechanistic indication that Est1 and Cdc13 might activate telomerase through exposing the telomere end for telomerase. Thus, results from this study are consistent with both recruiting and activation models that, in addition to recruiting telomerase to telomere, Est1-Cdc13 interaction also activates telomerase through change the accessibility of telomere end for telomerase extension. The interaction between Est1 and Cdc13 thus provides the second level of regulating telomerase activity through both recruiting telomerase and exposing the single-stranded telomeric DNA substrate. Through this simple mechanism, the fine-tuning of telomerase activity could be then achieved by other protein factors. For example, Stn1, Ten1, and Est3 could modulate the regulatory effects of Cdc13 and Est1 on telomerase^6^,^35^,^36^. The present *in vitro* system thus provides an excellent platform to further test directly the role of these proteins in regulating telomerase activity.

The mechanism of how Est1 alters the DNA-binding property of Cdc13 is not clear. Since the Cdc13-protected G-residues were exposed when Est1 was presented in DMS footprint analyses, it is possible that Est1 simply titrating Cdc13 off telomeres. However, judged by the tight binding affinity of Cdc13 to single-stranded telomeric DNA, with a K_d_ value of ~0.3–0.5 nM^37^,^38^, it is not likely that the relatively weak association between Est1 and Cdc13 (the K_d_ for Est1-Cdc13 is 250 nM^33^) would titrate away the binding between Cdc13 and telomeric DNA. Because the EMSA analyses from this study showed that Cdc13 remains bound to telomeric DNA with the addition of Est1 and it was reported by Zakian’s group that Est1 could form stable complex with Cdc13-DNA^33^, it is more likely that Est1 changes the conformation or rearrange the position of Cdc13 on the telomere to expose the telomeric end for telomerase extension.

It is interesting to note that the relieved telomerase activity can only be observed when Est1 is in excess (Figs 5 and 6). The need of using excess amount of Est1 in our analysis could be explained using the concept of fractional occupancy. Here, the fractional occupancy is defined as the fraction of Cdc13 that is bound by Est1. With the dissociation constant between Cdc13 and Est1 as 250 nM^33^, the calculated fractional occupancy values at 80 and 240 nM Est1 are 20% and 45%, respectively (Fig. S5). These values suggest that only 20% of Cdc13 is bound by Est1 when they are in equal molar ratio (80 nM Cdc13 and 80 nM Est1), and about half of Cdc13 is occupied by Est1 when Est1 is in 3-fold molar excess (80 nM Cdc13 and 240 nM Est1). This study showed that only ~40% of telomerase activity was restored at 240 nM Est1 (Figs 5 and 6). Significantly, these experimental values are in good agreement with the theoretical values. Thus, although Est1 is in excess, only a fraction of Est1 binds to Cdc13 to restore telomerase activity.

This study found that the binding of Cdc13 to telomeric DNA inhibits telomerase activity and the telomere-binding activity of Cdc13 is required for inhibition, which are consistent with a previous report by Zappulla *et al*.^24^. The present results and the findings of Zappulla *et al*. support a general mechanism that binding of a telomere-binding protein inhibited the access of telomerase to telomeres^24^. The conclusion is different from that reported by DeZwaan *et al*. that Cdc13 activated telomerase activity^26^. This study also showed that Est1 was capable of restoring telomerase activity on Cdc13-bound DNA to relieve the inhibitory effect of Cdc13, although Est1 alone did not affect telomerase activity. Again, the current findings are different from results reported by DeZwaan *et al*. that Est1 protein stimulated telomerase activity^25^. Although what accounts for the apparent discrepancy remains unclear, it may be due mainly to the ways telomerases were prepared. DeZwaan *et al*. used telomerase activities partially purified from column fractions of yeast extracts^25^,^26^. In reconstitution systems used by Zappulla *et al*., two telomerase activities were reconstituted. In the first system, the core telomerase enzyme containing Est2 and a short version of Tlc1 RNA was used. These two components were generated by *in vitro* transcription and translation system using rabbit reticulocyte lysates. Telomerase reconstituted through the *in vitro* translation system should not contain any yeast protein. The second system was prepared from yeast cells overexpressing Tlc1 RNA and ProA-tagged Est2 protein, similar to a system used in the current analysis. This study also reconstituted telomerase using two versions of Est2, an N-terminal GST-tagged and a C-terminal TAP-tagged, with full-length Tlc1 RNA. These reconstituted telomerases are functional and no Est1 or Cdc13 was detected. Reconstituted telomerases of Zappulla *et al*. and this study represent the core catalytic enzymatic activity of telomerase. In contrast, telomerase partially purified from yeast might behave as a holo-enzyme with associated proteins co-isolated with it^25^,^26^. Indeed, it was noted that Est1 is presented in telomerase preparations of DeZwaan *et al*.^25^. These associated proteins might affect telomerase activity that contributes to the apparent differences. It is also possible that the way Cdc13 and Est1 were expressed and isolated caused the differences. While DeZwaan *et al*. isolated both Cdc13 and Est1 from *E. coli*^26^, this study and Zappulla *et al*.^24^ expressed and isolated recombinant Cdc13 and Est1 from insect cells using the baculovirus expression system. This study also used a TAP-tagged system to express and isolate Est1 from yeast. Although less likely, this possibility cannot be fully ruled out. Nevertheless, from the perspective of defining the roles of Est1 and Cdc13 in telomerase regulation, the reconstituted system of this study might be advantageous over the partially purified protein in achieving this goal.

Regulation of telomerase activity by controlling the accessibility of telomere end appears to be a common mechanism among different species. In human cells, hPOT1 binds to the single-stranded telomeric DNA and inhibits telomerase from elongating the telomeres^39^,^40^. However, telomerase activity is fully restored when a > 6-nt tail is exposed beyond the hPOT1-binding site^40^. Thus, telomerase accessibility is modulated by positioning hPot1 on the single-stranded tail in human cells. Since telomerase activity was inhibited by Cdc13 on DNA with single-stranded tails ranging from 15–25 nucleotides^24^, the positioning of Cdc13 on telomeres might not be a major mechanism for regulating telomerase accessibility in yeast. This study found that the interaction between Cdc13 and G-residues on telomeric DNA was reduced by Est1. The reduced telomere-binding activity of Cdc13 might expose the telomere end for telomerase to extend telomere. Thus, the current results support a common mechanism of telomerase regulation by substrate accessibility.

## Methods

### GST-Est2 and Tlc1 RNA co-overexpression and affinity purification

Yeast strain BJ2168 harboring plasmids pRS424-GAL-TLC1 and pEG(KT)-EST2 was used as the host for telomerase expression^27^. A single colony was inoculated into the synthetic medium containing 3% raffinose and grown to OD_600_ = 0.7. Induction of GST-Est2 and Tlc1 RNA expression was achieved by addition of 3% galactose. After induction for 12 hr, yeast cells were harvested by centrifugation and re-suspended in lysis buffer (50 mM Tris-HCl pH 8.0, 150 mM NaCl, 1 mM EDTA, 0.4 mM PMSF, 10 mM dithiothreitol, and 40 units/ml RNasin). Total cell extracts were prepared by breaking the cells using a cell disruptor followed by centrifugation at 17,500 g. GST-Est2/Tlc1 RNP was then isolated by precipitation using 40% ammonium sulfate and affinity binding using glutathione-sepharose beads (Sigma). The protein complexes were eluted with buffer containing 50 mM Tris/HCl, pH 8.0, 1 mM EDTA, 5% glycerol, 0.01% Triton X-100, 150 mM NaCl, 0.0005% octanol, 20 mM glutathione, 5 mM DTT, and 40 units/ml RNasin, divided into aliquots, and stored at −70 °C.

### Purification of TAP-tagged telomerase

Plasmid pRS426-GAL-EST2-TAP carrying TAP-tagged Est2 was transformed into yeast strain CY123 harboring plasmid pRS424-GAL-TLC1 (from Dr. Jin-Qiu Zhou, Chinese Academy of Sciences, China). The resulting yeast cells were grown in medium containing 2% raffinose at 30 °C until OD_600_ to 1.0. Galactose was then added to the cells (final concentration 3%) to induce telomerase expression, followed by culture for another 12–16 hr. The TAP-tagged telomerase was purified following protocol described by Puig *et al*.^41^. The eluted proteins were divided into aliquots and frozen directly using a dry ice/ethanol bath.

### DNA substrates

Oligonucleotides T21 (5′-ATGGCTAGGTAGCCGAATTGCGTAGGTGGGTGTGGTGTGTGTGGG-3′) and C20 (5′CCCACCTACGCAATTCGGCTACCTAGCCAT-NH_2_-3′) were used in this study. To prepare the tailed-duplex DNA substrates, T21 DNA was mixed with excess amount of unlabeled C20 DNA and incubated at 100 °C for 5 min. DNA mixtures were then slowly cooled at room temperature and subsequently separated by a native 8% polyacrylamide gel to isolate the tailed-duplex DNA substrate.

### Primer extension assay

Telomerase reactions were carried out in 30 μl of reaction buffer (10 mM Tris-HCl, pH 8.0, 1 mM DTT, 1 mM spermidine, 2 mM Mg-acetate, and 50 μM dTTP) with 30 nM DNA substrate and 10 μCi [α-^32^P]dGTP at 30 °C for 1 hr. The reactions were stopped by addition of 80 μl of extension-stopping solution (10 mM Tris-HCl, pH 8.0, 20 mM EDTA, and 0.1 mg/ml RNase A) at 30 °C for 10 min, followed by addition of 100 μl of SDS/PK solution (10 mM Tris-HCl pH 8.0, 0.5% SDS, and 0.3 mg/ml proteinase K) at 37 °C for 30 min. The reaction products were extracted by phenol/chloroform, and then precipitated with 7.5M NH_4_OAc and 2.5 volumes cold 100% ethanol. The reaction products were then separated by a 12% sequencing gel and then visualized using a PhosphorImager (Molecular Dynamics). To quantify telomerase activity, the total intensity of all major bands (+1 to +7) was measured for each reaction (ImageQuant). Quantifications were obtained from the average of three-six independent experiments. The mean values of the results are plotted and the standard deviations are plotted as error bars.

### Tethered-Particle Motion (TPM)-based *in vitro* telomerase activity assay

The single-molecule telomerase assay was conducted by following a protocol that was developed by Li *et al*.^30^. Briefly, a tailed duplex DNA substrate formed by digoxigenin-labeled 5′-ATTCCTTGCGGCGGCGGTGCTCAACGGCCTCAACCTACTACTGGGCTGCTTCCTAATGCAGGAGTCGCATAAGGGAGAGCGTCGACCGATGGTGTGTGTGGG-3′ and 5′-ATCGGTCGACGCTCTCCCTTATGCGACTGGTGCATTAGGAAGCAGCCCAGTAGTAGGTTGAGGCCGTTGAGCACCGCCGCCGCAAGGAAT-3′ (1 nM) was first incubated at 30 °C overnight with 4 nM of purified telomerase in the presence or absence of Cdc13. The extension reactions were stopped by introducing RNase H and Proteinase K (NEB) at 30 °C for 20 min followed by heating at 80 °C to disrupt telomerase-DNA interaction. The reaction products were then loaded onto anti-digoxigenin (20 ng/μL)-coated slides. To visualize the extent of telomerase-mediated telomere lengthening, immobilized reaction products were incubated with 5′ biotinylated oligonucleotide probe (5′-CCCACACACACC-3′, 5 μM) for 20 min. Streptavidin-coated polystyrene beads were then introduced into the reaction chamber for tether visualization. The sequence of oligonucleotide probe is complementary to the telomerase-extended DNA; hence, the oligonucleotide probe could be annealed to several different positions in the case of multiple runs of telomere extension. Single-molecule tethering events were next observed under an inverted microscope (Olympus IX-71) with differential interference contrast (DIC) image method. The imaging acquisition was at 30 Hz. The Brownian motion amplitude (BM) was defined as the standard deviation of the centroid position of the tethers with an average of 40 frames, and the BM values were drift-corrected using a stuck bead pre-adsorbed on the surface. Quantifications were obtained from the average of three independent experiments.

### Expression and purification of 6xHis-tagged Cdc13, Est1, and Cdc13(451-693)

The baculovirus system was utilized to purify Cdc13 and Est1^29^. Insect cell line sf21 was used as the host for virus propagation and protein purification. *E. coli* BL21(DE3) pLysS was used as the host for Cdc13(451–693) purification^29^. A TAP-tagged Est1 was also isolated following the protocol described by Puig *et al*.^41^

### Electrophoretic mobility shift assay (EMSA)

Oligonucleotides was first 5′ end labeled with γ-^32^P-ATP (3000 mCi/mM, NEN) using T4 polynucleotide kinase (New England Biolabs) and subsequently purified from a 10% sequencing gel after electrophoresis. To prepare the tailed-duplex DNA substrates, the 5′ end-labeled T21 DNA was mixed with excess amount of unlabeled C20 DNA to form the tailed-duplex DNA substrate described earlier. EMSA experiments were conducted on 6% non-denaturing polyacrylamide gels. To quantify DNA-binding activity, the fraction of free probe was measured for each reaction (ImageQuant). The values were obtained from three independent experiments. The mean values of the results are plotted and the standard deviations are plotted as error bars.

### Dimethyl Sulfate Footprint

Radiolabeled DNA was mixed with Cdc13 and/or Est1 in 50 mM cacodylate buffer, pH 7.0, and incubated at 25 °C for 10 min. Dimethyl sulfate (DMS) was added to a final concentration of 0.05% and incubated at 22 °C for another 15 min. The mixtures were ethanol-precipitated, resuspended in 50 μl of 10% piperidine, incubated at 95 °C for 15 min, and separated by 10% denaturing gels. The gels were dried and analyzed with a PhosphorImager (Molecular Dynamics). To quantify the footprints, each lane was divided into 400 sections. The radioactivity was measured using ImageQuant and the relative radioactivity levels were then plotted.

## Additional Information

**How to cite this article**: Chen, Y.-F. *et al*. Modulation of yeast telomerase activity by Cdc13 and Est1 *in vitro.* *Sci. Rep.* **6**, 34104; doi: 10.1038/srep34104 (2016).

## Supplementary Material

Supplementary Information
