# Supplementary material for: Modulation of yeast telomerase activity by Cdc13 and Est1 in vitro
Source: Sci Rep. 2016 Sep 23;6:34104. doi: 10.1038/srep34104 (PMC5034320; doi:10.1038/srep34104)
Supplement: Supplementary Information [file srep34104-s1.pdf]

Modulation of yeast telomerase activity by Cdc13 and Est1 *in vitro*

Supplementary figures

Yu-Fan Chen[1], Chia-Ying Lu[2], Yi-Chien Lin[1], Tai-Yuan Yu[1], Chun-Ping Chang[2], Jing-Ru Li[3], Hung-Wen Li[3], and Jing-Jer Lin[1,2,*]

[1] Institute of Biopharmaceutical Sciences, National Yang-Ming University, Taipei 112, Taiwan

[2] Institute of Biochemistry and Molecular Biology, National Taiwan University College of Medicine, Taipei 100, Taiwan

[3] Department of Chemistry, National Taiwan University, Taipei 100, Taiwan

•Corresponding author: Jing-Jer Lin, Institute of Biochemistry and Molecular Biology, National Taiwan University College of Medicine, Taipei 100, Taiwan. E-mail: jingjerlin@ntu.edu.tw

Running title: Cdc13 and Est1 modulates telomerase activity

Key words: telomerase, telomere, yeast, Cdc13, Est1

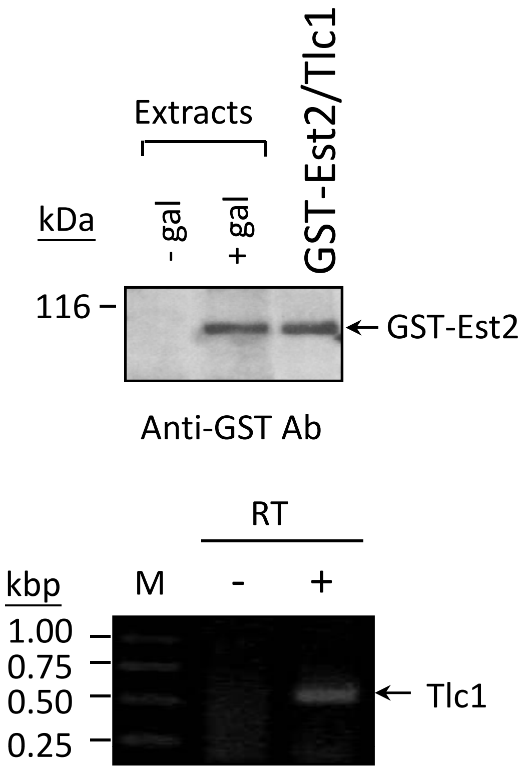

Figure S1. Both GST-Est2 and Tlc1 RNA are presented in the purified GST-Est2/Tlc1 RNP. Total extracts prepared from un-induced and galactose-induced yeast cells and the isolated GST-Est2/Tlc1 RNP was separated by an 8% SDS-polyacrylamide gel. GST-Est2 was detected by immunoblotting analysis using polyclonal anti-GST antibodies (top panel). Positions of the GST-fused Est2 were indicated. Tlc1 RNA was detected by RT-PCR in the isolated GST-Est2/Tlc1 RNP (bottom panel).

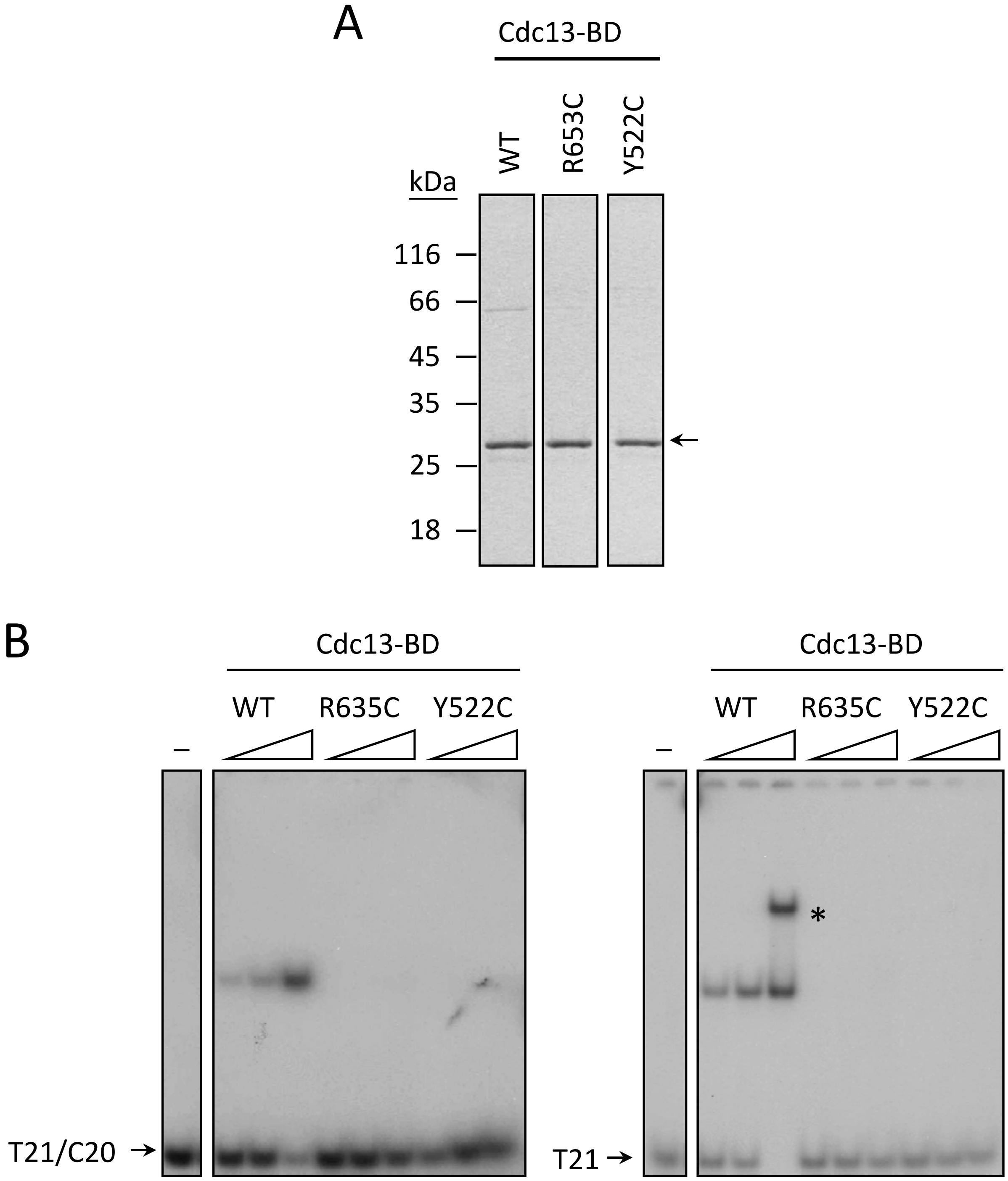

Figure S2. (A) Purification of Cdc13(451-693) proteins. The DNA binding domain (Cdc13-BD) of wild-type and two telomeric DNA binding-defective mutant proteins, Cdc13^{R653C}and Cdc13^{Y522C}, were expressed and purified from *E. coli* cells. Two μg of purified proteins was analyzed on a 12% SDS polyacrylamide gel. The Coomassie blue-stained gel is shown. (B) The telomeric DNA binding activity of wild type and mutant Cdc13(451-693) proteins. Thirty nM of ^{32}P-labeled T21/C20 (left) or T21 (right) DNA was mixed with 20, 40, and 80 nM of the purified proteins at room temperature for 5 min and analyzed by EMSA. Asterisk indicates the position for binding of a second Cdc13-BD molecule on T21 DNA.

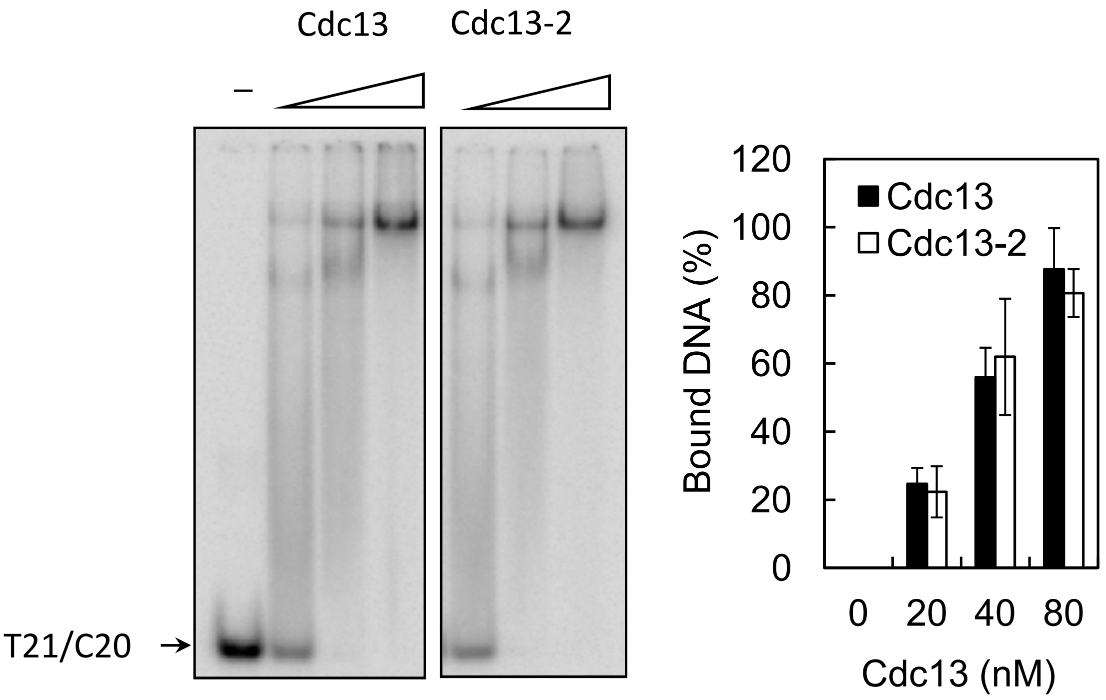

Figure S3. The DNA binding activity of Cdc13 and Cdc13-2. The DNA binding property of Cdc13-2 was analyzed by EMSA. The concentrations of Cdc13 and Cdc13-2 used in each set of experiments were 20, 40 and 80 nM.

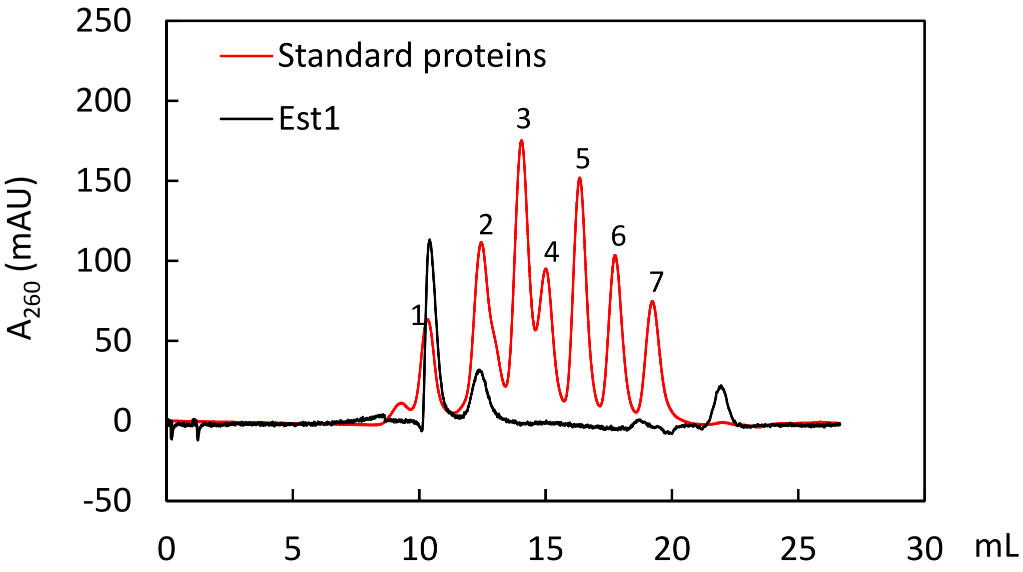

Standard proteins:

1. Ferritin (Mr. 440,000)
2. Aldolase (Mr. 158,000)
3. Conalbumin (Mr. 75,000)
4. Ovalbumin (Mr. 44,000)
5. Carbonic anhydrase (Mr. 29,000)
6. Ribonuclease A (Mr. 13,700)
7. Aprotinin (Mr. 6,500)

Figure S4. Gel filtration column chromatography of Est1. The isolated Est1 was loaded onto Superdex 200 column. The eluted protein fractions were monitored using absorption at 260 nm.

For Cdc13 and Est1 interaction:

$$[\text{Cdc13}] + [\text{Est1}] \longrightarrow [\text{Cdc13-Est1}]$$

$$K_d = \frac{[\text{Cdc13}]_{free} \times [\text{Est1}]_{free}}{[\text{Cdc13-Est1}]}$$

Here: $[\text{Cdc13}]_{total} = [\text{Cdc13}]_{free} + [\text{Cdc13-Est1}]$

$[\text{Est1}]_{total} = [\text{Est1}]_{free} + [\text{Cdc13-Est1}]$

Thus,

$$K_d = \frac{([\text{Cdc13}]_{total} - [\text{Cdc13-Est1}])([\text{Est1}]_{total} - [\text{Cdc13-Est1}])}{[\text{Cdc13-Est1}]}$$

The above equation can be rewritten as:

$$[\text{Cdc13-Est1}]^2 - ([\text{Cdc13}]_{total} + [\text{Est1}]_{total} + K_d)[\text{Cdc13-Est1}] + [\text{Cdc13}]_{total}[\text{Est1}]_{total} = 0$$

Next, solve the equation using quadratic formula:

$$[\text{Cdc13-Est1}] = \frac{([\text{Cdc13}]_{total} + [\text{Est1}]_{total} + K_d) - \sqrt{([\text{Cdc13}]_{total} + [\text{Est1}]_{total} + K_d)^2 - 4[\text{Cdc13}]_{total}[\text{Est1}]_{total}}}{2}$$

Thus, when $[\text{Cdc13}]_{total}$ is 80 nM, $[\text{Est1}]_{total}$ is 240 nM, and K_d = 250 nM, the [Cdc13-Est1] is calculated to be 36 nM.

$$\text{The fractional occupancy of Est1 on Cdc13} = \frac{[\text{Cdc13-Est1}]}{[\text{Cdc13}]_{Total}} = \frac{36}{80} = 0.45$$

In a different condition, $[\text{Cdc13}]_{total}$ is 80 nM, $[\text{Est1}]_{total}$ is 80 nM, the [Cdc13-Est1] is calculated to be ~16 nM.

$$\text{Fractional occupancy} = \frac{16}{80} = 0.20$$

Figure S5. Calculation of Est1 fractional occupancy
